# Pelvic Peritonitis*Read at Palestine meeting of the East Texas Medico-Chirurgical Society, May 31, 1901.

**Published:** 1901-10

**Authors:** W. C. Lipscomb

**Affiliations:** Crockett, Texas


For Texas Medical Journal.

### Pelvic Peritonitis.*

BY W. C. LIPSCOMB, M. D., CROCKETT, TEXAS.

The important functions of the female pelvic organs render this

*Read at Palestine meeting of the East Texas Medico-Chirurgical Society, May 31, 1901.

locality one of great importance to the general practitioner. Nothing taxes his powers of discrimination more than the various and serious maladies which he is here called upon to consider, and perhaps nothing yields so rich a reward to timely and judicious treatment.

In referring to the anatomy of the parts it is necessary to say only that this serous membrane is peculiar in being continuous with the mucous membrane of the uterus through the Fallopian tubes. This is important in consideration of causes. The process of inflammation is the same here as in other serous membranes. A stage of congestion, then one of effusion, and finally one of resolution and absorption. This stage may be complete or partial, leaving more or less organized lymph behind, forming permanent adhesions, and by a surprising degree of contraction often producing most serious displacements. Suppuration may occur also. The pus usually gathers in Douglas *cul de sac* and becomes circumscribed here by virtue of plastic adhesions thrown out for the purpose. It may form also in the tubes and be circumscribed, there forming pouches. Another form of the disease is attended with very little, if any, effusion, no pus, and is analagous to dry pleurisy.

Pelvic peritonitis does not tend to become general and must not be confounded with that form originating in uterine inflammation after labor and rapidly becoming general.

Acute pain in the hypogastrium with marked tenderness on pressure usually sounds the danger signal. The patient is usually found to have a temperature of 103 to 106°, and a pulse of 110 to 120, small and wiry. There is frequently vomiting of bile and constipation. There is marked tympanitis, and sometimes the intestinal coils can be plainly felt on gentle palpation. The urine is scanty and passed with much pain or not at all. The facial expression is indicative of suffering and anxiety. Skin hot and dry. Decubitus on back with thighs flexed. After the third day vaginal examination reveals thickening of the roof of the pelvis and fixation of the uterus and its appendages. The tumefaction is high up in distinction to that of cellulitis, which is low and lateral, while hematocele generally occupies the Douglas *cul de sac.* In cellulitis we contrast low fever, dull pain, slight disturbance of the digestive system and absence of marked tympanitis with high fever, sharp lancinating pain, marked disturbance of the digestive system and marked tympanitis and thigh flexion. In hematocele we have in the beginning shock with slow, weak pulse and subnormal temperature.

Predisposing causes of pelvic peritonitis are functional and organic disturbances of the uterus and ovaries, chronic affections followed by traumatisms in the treatment of them. It may occur secondary to cellulitis, hematocele, tumors taking on inflammation, etc. The most common source of infection is through the tubes. This may be of the nature of a discharge from the inflamed endometrium, retained decidua or other putrefactive substances, but most commonly the gonorrheal virus. So, in establishing our plan of treatment we must not lose sight of a probable active source of infection and the value of its removal. Give the patient at once one-fourth grain of morphia hypodermically. If there is reason to suppose the uterus contains infectious matter introduce the speculum. A turgid appearance with a purulent or shreddy discharge issuing from the os will warrant the use of the douche. If the os is narrow use the metal dilator, expanding in three diameters, until the douche can be accommodated with room for a free return current. Then introduce the douche until the fundus is reached and run in about a quart of a 1-2000 solution of bichloride of mercury as warm as patient will allow. All this, of course, must be conducted with such gentleness as not to give rise to the slightest traumatism or excite the patient physically or mentally.

The morphia injections should now be repeated as often as necessary to keep the patient easy, the bowels constipated and the inflammatory process in abeyance. Of other methods of administering opiates perhaps the deodorized tincture might be best borne. Small doses of aconite, carefully noting its action on the pulse, is useful in circumscribing the inflammatory process. Absolute quiet in every way must be enjoined strictly. If necessary remove the urine with the catheter. Keep hot turpentine fomentations, well covered with oil silk, applied constantly. Take care not to use the counter-irritation too severe in the beginning, and not to change local applications so often as to disturb the patient unnecessarily. When acute symptoms have subsided blistering the hypogastrium with tincture of iodine or cantharidal plaster will aid in absorption of plastic material.

The patient must be well sustained with nourishing diet, strychnia and quinine. As convalescence sets in the iodide of potassium with the red iodide of mercury will aid absorption.

The patient must now be guarded most carefully against too violent exercise, especially about the monthly period, jolting, sexual intercourse, etc., for fear the disease will be re-established.

If pus has formed and can be definitely located a free incision should be made. If the location is doubtful an exploratory puncture should be made, and the pus, if found, drawn off, the cavity washed with a 1-2000 bichloride solution and afterward with sterile water.

